# Differential Diagnosis and Treatment of Itching in Children and Adolescents

**DOI:** 10.3390/biomedicines9080919

**Published:** 2021-07-30

**Authors:** Seok-Young Kang, Ji-Young Um, Bo-Young Chung, Jin-Cheol Kim, Chun-Wook Park, Hye-One Kim

**Affiliations:** Department of Dermatology, Kangnam Sacred Heart Hospital, Hallym University, Seoul KS013, Korea; tjdjrdud@naver.com (S.-Y.K.); ujy0402@hanmail.net (J.-Y.U.); victoryby@naver.com (B.-Y.C.); aiekfne@naver.com (J.-C.K.); dermap@hanmail.net (C.-W.P.)

**Keywords:** children, adolescents, itching, pruritus, differential diagnosis, treatment of itching

## Abstract

Itching is prevalent in children with skin disorders and associated with effects on their mood, quality of life, and social functioning. Surprisingly, there are no data on childhood prevalence of pruritus in the general population. The aim of this article is to explore the epidemiology, clinical manifestation, and treatment for itch (pruritus) in the pediatric population (from infancy to adolescence), and to be helpful to primary care physicians who assess and diagnose pediatric patients with itching. In this study, we searched for specific keywords using PubMed and MEDLINE (Ovid) and, then, refined the retrieved searches for each cause and treatment. As a result of reviewing the literature, atopic dermatitis was shown to be the most common cause of itching, especially during infancy and through preschool. Not only skin disorders but also systemic diseases, drugs, and postburn states can predispose an individual to itching in childhood. There are traditional and newly developed treatment modalities for itching in pediatric patients. However, because the pharmacokinetics and pharmacodynamics of childhood are different from those of adults, the medications for itching have to be applied carefully for these age groups. There are many areas to be elucidated regarding the prevalence and objective assessment of pruritus in pediatric patients. Moreover, the safety profiles of medications in the pediatric population need to be better understood. Further studies to investigate itching in childhood are warranted.

## 1. Introduction

Pruritus (itch, or more commonly, itching) is prevalent in children with skin disorders such as atopic dermatitis, and is associated with effects on mood, quality of life, sleep, scholastic performance, and social and family functioning [1,2]. Itch is common in children; therefore, it is surprising that there are no data on the childhood prevalence of pruritus in the general population [3]. Most previous articles are related to specific diseases and usually include case reports. The aim of this article is to aid primary care physicians in effectively assessing and diagnosing most children and adolescents with itching. It also provides counseling for pediatric patients with itching and updates the status of local and systemic therapies. PubMed and MEDLINE (Ovid) were searched for the keywords: children, adolescents, and itching or pruritus for the interval from 2010 to 2021. We refined our search for each cause and treatment by adding appropriate keywords and performed a follow-up search through the bibliographies of the retrieved literature.

Itching in childhood is mainly associated with skin diseases. Systemic disease and drug reactions are rare compared to adults. Itchy skin conditions in children include eczema (particularly atopic dermatitis), rashes, infections/infestations, urticaria/mastcytosis, autoimmune disorders, and hereditary dermatoses [4]. For dermatologic treatment (topical or systemic) in children, dermatologists must take into account the physiology, and pathophysiology, and also pharmacokinetics and pharmacodynamics, which differ from those of adults [4]. The high ratio of body surface to body weight determines the absorption of topically applied drugs and emollients. Infantile skin is also characterized by a thinner epidermis and stratum corneum, and smaller corneocytes [5]. We should take these facts into account in the treatment of infants in general, particularly in those suffering from diseases with a known barrier defect such as atopic dermatitis (AD) [5].

Childhood medical care differs from adulthood in many ways. Proper contact with both children and parents is required to obtain reasonable statements during anamnesis. Clinical examination in children differs from that of adults. In the literature there are some itch assessment scales that may be used for childhood cases, for example, the Itch Assessment Scale for the Pediatric Burn Survivor [6]. This Itch Man Scale correlates with other scales such as the 5D Itch Scale and the Visual Analogue Scale [7]. In this article, the authors describe the clinical features and treatment of itching related to atopic dermatitis, psoriasis, urticaria, chickenpox, and other skin and systemic conditions. These are common itching disorders in childhood and adolescence.

## 2. Common Disorders in Children and Adolescents That Cause Itching

**Figure 1:**
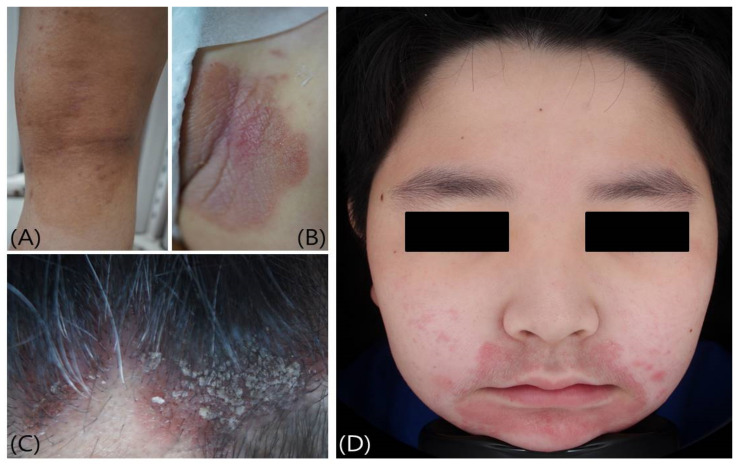
Common disorders in children and adolescents that cause itching: (**A**) Atopic dermatitis, (**B**) Psoriasis, (**C**) Seborrheic dermatitis, and (**D**) Contact dermatitis.

Itching in young children can be caused by a variety of skin diseases (Figure 1), and the cause of the disease varies according to age (Table 1) [8,9,10,11,12]. Atopic dermatitis is most common during infancy and preschool. Contact dermatitis, atopic dermatitis, and acne vulgaris are common skin diseases in both school and adolescent groups.

**Figure 2:**
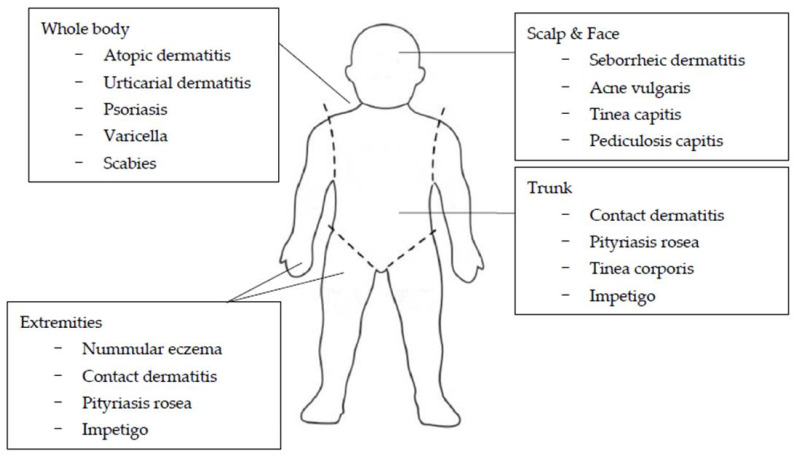
Common areas of skin diseases with itching in children and adolescents.

The regional distribution and morphologic configuration of cutaneous lesions provide important clues for the diagnosis of various dermatologic disorders (Figure 2) [13,14,15,16]. Lesions that involve the scalp and face include seborrheic dermatitis, acne vulgaris, tinea capitis, and pediculosis capitis [15]. Seborrheic dermatitis is an erythematous, scaly or crusting eruption that occurs characteristically on the scalp, face, postauricular, presternal, and intertriginous areas [14,15]. Morphological and topographical variations occur in varying combinations and with varying degrees of severity, ranging from mild involvement of the scalp with blepharitis to generalized and sometimes severe erythematous scaling eruptions. Acneiform lesions are lesions with the form of acne, and an acneiform distribution refers to lesions that appear mainly on the face, neck, chest, upper arms, shoulders, and back [13,14,15].

Many diseases can affect the trunk as well. Contact dermatitis, pityriasis rosea, tinea corporis, and impetigo are common examples [16]. Pityriasis rosea begins as a solitary round or oval scaling lesion known as the herald patch in 70% to 80% of cases, which may be annular and is often misdiagnosed as tinea corporis [16]. After an interval of several days to two weeks, affected individuals develop a generalized symmetrical eruption involving primarily the trunk and proximal extremities [16]. A clue to diagnosis is the distribution of the lesions, with the long axis of these oval lesions parallel to the lines of cleavage in what has been termed a Christmas-tree pattern. A common variant, inverse pityriasis rosea, is often localized in the inguinal region, but the parallel nature of the long axis of lesions still remains characteristic [16].

Certain diseases, such as nummular eczema, contact dermatitis, pityriasis rosea, and impetigo, generally involve the extremities. The lesions of erythema multiforme may be widespread, but there is a distinct predilection for hands and feet (particularly in the palms and soles) [16].

Atopic dermatitis, urticarial dermatitis, psoriasis, varicella, and scabies tend to affect the whole body. Sites of predilection for atopic dermatitis include the face, trunk, and extremities in young children. On the other hand, the antecubital and popliteal fossae are the most common sites in older children and adolescents [16]. Lichen planus often affects the limbs. Favorite areas are the lower extremities, the flexor surface of the wrists, the buccal mucosa, the trunk, and the genitalia. Although exceptions do occur, lesions usually appear in a bilaterally symmetric pattern favoring the elbows, knees, and scalp, as well as the lumbosacral, perianal, and genital regions. Nail involvement, characterized by pitting of the nail plate, discoloration of nail, nail plate separation from the nailbed (onycholysis) and accumulation of excessive subungual scale (subungual hyperkeratosis), is a useful diagnostic sign. Scabies typically affects the wrists and hands (especially interdigital webs), forearms, areolae, genitalia, and buttocks in older children and adolescents. Other family members may complain of itching in similar areas. However, because the sites of predilection differs, the diagnosis is often missed in infants and young children. Areas typically involved in infants and young children include palms, soles, and often the head and neck. Disappearance of burrows, which are characteristic primary lesions for scabies, is particularly common in infants due to strong hygienic measures, excoriation, eczematization, crusting, and secondary infection [16]. Additionally, herpes simplex may involve anywhere on the body, but the lips, face, and genitalia are clearly more commonly involved [16].

### 2.1. Atopic Dermatitis

Atopic dermatitis (AD) is a common chronic pruritic skin disease especially in children, and is associated with a personal and family history of atopy. AD is relatively common, affecting 10% to 20% of children in developed countries. AD is an inflammatory dermatosis with the hallmark of intense itching. Children with moderate-to-severe AD suffer from intolerable itching and disruption of sleep and concentration, as well as social stigmatization. Although it is generally known that the intensity of pruritus in AD is severe, there are few studies directly comparing the intensity of pruritus in AD with other diseases. In one study, 148 patients with chronic dermatological pruritus were enrolled to compare the intensity of pruritus using a newly developed scale: the 12-Item Pruritus Severity Score (12-PSS) [17]. In this study, AD was the most pruritic skin disease, followed by psoriasis and lichen planus. In another meta-analysis, the intensity of pruritus in AD patients showed no significant difference in mean baseline itch scores than in patients with moderate-to-severe psoriasis, though psoriasis is traditionally thought to be less pruritic than AD [18]. The assessment of pruritus in an individual child is also a problem due to the subjective nature of the itching sensation. Like adults, the SCORAD index, the Eczema Area and Severity Index (EASI), and the investigator global assessment (IGA) are still the most commonly used to assess the intensity of AD in childhood and adolescence, although there are limitations to assessing them accurately from the anamnesis of very young patients [3,19]. The classic lichenifications in the folds of the extremities, which are associated with permanent scratching, appear in later childhood. AD begins within the first 5 years of life in 90% of patients, usually presenting with a characteristic age-dependent distribution with facial, scalp, and extensor involvement in infants and young children, and predominant flexural involvement in older children and adults. Pruritus xerosis is a common feature in children with AD. There are many known factors that cause atopic dermatitis. One of these factors is a contact allergen. De Waard-van der Spek and Oranje [20] showed that 55% of children with positive reactions for one or more contact allergen had atopic dermatitis. They recommended that there are indications for patch testing in children with atopic dermatitis, especially if they are resistant to treatment modalities. The objectives of atopic dermatitis management are to improve patients’ quality of life and prevent infectious complications, while minimizing potential drug side effects. The best way to control all aspects of AD morbidity including pruritus is through skin hydration, recovery of the skin barrier function, and control of skin inflammation [21]. This may result in the prevention of re-exacerbation of the disease and steroid-sparing benefits. It also helps relieve the itch sensation. Standard therapy for patients with atopic dermatitis includes topical anti-inflammatory drugs (topical corticosteroids and calcineurin inhibitors) in addition to the use of moisturizers [2,21]. They are used in both reactive and prophylactic treatment to control skin inflammation. Although topical corticosteroids are still the treatment of choice for the management of atopic dermatitis in children, calcineurin inhibitors are increasingly used as well [2]. Calcineurin inhibitors are recommended for use on sensitive areas such as the face, folds, and genitoanal areas, and provide antipruritic effects. Recently, it has been emphasized once again that calcineurin inhibitors are safe for the treatment of children. Cyclosporine (licensed from 16 years on) is the drug used on the label; mycophenolate mofetil, methotrexate, and azathioprine are drugs used off-label. All have antipruritic properties [2]. Recently, a Canadian research team pointed out the excellent antipruritic effect of clonidine (adrenergic agonist) and trimeprazine (phenothiazine) combination therapy in a 6-year-old boy suffering from severe atopic dermatitis and intractable pruritus. There are also other interesting antipruritic drugs in development [2]. Draelos et al. [22] showed the positive effect of the topical phosphodiesterase-4 inhibitor Crisaborole in children and adolescents with mild-to-moderate atopic dermatitis based on data from four studies. A 4-point rating was used to rate the severity of pruritus. Treatment showed a statistically significant decrease in pruritus severity. Moreover, many new drugs, especially biologics, are in the clinical trial phase [23]. Along with the severity score, the severity of pruritus has also been assessed. Adult studies lead to child studies. For example, studies with the interleukin (IL)-4/IL-13-receptor-α dupilumab are already underway in children. An obsession with the details of the treatment plan, regular follow-up to adjust treatment plans for moderate-to-severe cases, and extensive education at each visit are absolutely important for successful management. Multidisciplinary treatment teams will help manage moderate-to-severe cases [2].

### 2.2. Nummular Eczema

Nummular eczema is a chronic inflammatory condition characterized by pruritus and histologically characterized by spongiosis [24]. A very special form in childhood is nummular eczema, which may be very exudative and is seen not only at the extremities but also on the trunk compared to adults [25]. Thus, atopic dermatitis and nummular eczema in infants and children assumes different clinical expressions compared with adults. Chronic nummular eczema, in contrast to the acute phase, is recognized by lesions that are dry and leathery, and the term “lichenification” is applied to these to indicate that the linear markings of the skin at the site of the plaques stand out prominently and induces severe pruritus [25]. Management of nummular eczema includes general measures aimed at reducing skin dryness and exposure to irritants and treating skin inflammation. High- potency topical corticosteroids are the first-line therapy for nummular eczema [25]. Topical corticosteroids are applied 1 to 2 times daily for 2 to 4 weeks or until the lesions disappear. The use of occlusive dressings may improve the penetration of corticosteroid into the skin and may lead to a faster response. Patients with extensive disease that does not respond to topical corticosteroids can be treated with narrowband ultraviolet B (NB-UVB) therapy [26]. In general, it takes 10 to 30 treatments 2–3 times a week for a response [27]. Once all lesions have been removed, the frequency may be reduced, to once weekly for a month, then to every other week for two months, as needed and tolerated. For patients with refractory disease, short-term systemic corticosteroids are an alternative treatment option when phototherapy is not available or is not possible for logistical reasons [27]. For clinicians who prefer oral corticosteroids, prednisone can be started at 40 mg per day, reduced by 10 mg every five days and then discontinued. Systemic corticosteroids, methotrexate, or cyclosporine for the treatment of nummular eczema have not been evaluated in clinical trials. Their use is based on evidence of efficacy for the treatment of other severe dermatitis. The use of methotrexate for nummular eczema was reported in two series of 25 and 28 pediatric patients. Complete remission was observed in 16 and 10 children after a mean treatment period of 10.5 months, respectively [28].

### 2.3. Urticaria

Urticaria is defined as the sudden appearance of very itchy wheals that usually last from a few hours up to 24 h. It is estimated that up to 25% of adults will experience at least one episode of acute urticaria sometime in their lifetime, and only around 3% will develop chronic spontaneous urticaria. In children, urticaria appears to be less common [28]. The cumulative prevalence of urticaria in children up to the age of 10 years was 14.5% for boys and 16.2% for girls. In a European research study, the reported incidence of all forms of childhood urticaria was ~3.4–5.4% [28]. The prevalence of chronic urticaria, defined as urticaria lasting more than six weeks, is estimated to affect 0.1–0.3% of children in the United Kingdom [28]. Within the cohort of children with chronic urticaria, reports of the successful discovery of the etiology ranged from only 48/226 of children (21%) aged 1–14 years old to 19/44 children (43%) up to 14 years old, to 90/163 children (55%) aged from six months to 16 years [28]. Infection also appears to play a more important role in both infants and children compared to adults, suggesting that urticaria in children is not the same disorder as in adults. Urticaria is classified into the following types based on duration and etiology: spontaneous acute urticaria, spontaneous chronic urticaria, physical urticaria, and others [28]. These types require different management. The disease is very common in childhood and is most often manifested as an acute urticaria (lasting less than 6 weeks). Often, this is related to a viral infection. In a study of 54 children with urticaria, Sackesen et al. [29] found that 68.5% suffered from the acute form. In about half of these patients, infection was the cause (48.6%), whereas in the group with chronic urticaria, physical factors were the main cause (52.94%). The study recommended the diagnostic measures for specific infectious agents as well as detailed history. The documented identification rate for causes in children with chronic urticaria in the study is 20–50%. However, in 30–47% of these children, urticaria is associated with a form of autoreactivity (confirmed by a positive autologous serum skin test) induced by IgG antibodies to the high-affinity IgE receptor (FcεRIα) [30,31]. Second-generation antihistamines are the main pharmacological treatment for children with urticaria. Omalizumab is recommended for recalcitrant cases, particularly in children with autoreactive chronic urticaria.

### 2.4. Mastocytosis

Mastocytosis in pediatric patients is typically manifested as mastocytoma and urticarial pigmentosa. Although there are no clear data about the prevalence of cutaneous mastocytosis, it was reported in 1/500 children presenting to pediatric dermatology clinics in one study [32]. It is clinically manifested as urticaria pigmentosa, diffuse cutaneous mastocytosis or solitary mastocytosis. Other forms (bullous, diffuse), including the involvement of extracutaneous organ systems such as the gastrointestinal tract, skeletal, and bone marrow, are rare compared to adults [16]. Diagnosis is made by morphology and positive Darier sign. Infants in particular show a tendency to respond strongly to mechanical stimuli of Darier sign, which can lead to flushing as well as the formation of urticaria and bullae. In addition to mechanical triggers, temperature extremes (heat, cold), exercise, foods, drugs, and venoms may also trigger mast cell degranulation. Histologically, within the macules and plaque, mast cells are predominantly located in the papillary dermis. Mast cells are round or spindle-shaped with abundant eosinophilic cytoplasm, distinct cytoplasmic boundaries, and large, pale nuclei. Eosinophils are often present and edema of papillary dermis and subepidermal vesiculation are also observed [16]. Bullous mastocytosis may be diagnosed with a Tzanck smear; infiltration may be mild and perivascular. In telangiectasia macularis eruptive perstans, features may be subtle, with an increase in mast cells around dilated superficial capillaries, basal cell hyperpigmentation of overlying epidermis, and superficial lymphohistiocytic infiltration. There is a useful scoring system (SCORMA) that correlates with serum tryptase level and includes surface area involvement and intensity of the lesions as well as subjective symptoms such as pruritus [32,33]. Compared to adults, children have a very good prognosis and remission is very common. Therapy is symptomatic, including the avoidance of triggers and administration of Type 1 antihistamines [32,33].

### 2.5. Seborrheic Dermatitis

Seborrheic dermatitis occurs in the pediatric population and is most commonly seen in infants between the ages of three weeks and 12 months, and in adolescents. It has been reported in approximately 10% of infants younger than one month [34]. The prevalence peaks at the age of three months (~70%) and decreases steadily in the following months, affecting ~7% of children aged one to two years. Research to elucidate the prevalence and severity of pruritus is lacking, to our knowledge. However, it is empirically known that the pruritus of children with seborrheic dermatitis is slight or even absent [35]. Seborrheic dermatitis occurs in areas with abundant sebaceous glands, such as the scalp, nose, and back. Seborrheic dermatitis in infants usually appears on the scalp and is commonly known as “cradle cap.” In older children and adults, seborrheic dermatitis on the scalp is commonly referred to as “dandruff.” The exact cause of seborrheic dermatitis is unknown, but it is believed that factors that cause the skin to overproduce oil, such as genes and yeast, that naturally inhabit the skin, stress, chemical stimuli, dry and cold weather, etc., act in a complex way. In infants, researchers believe seborrheic dermatitis is triggered in part by maternal hormones [34]. Sometimes, seborrheic dermatitis appears on the face of infants, especially around the eyes and nose. It can also appear in the diaper area and in the folds of babies’ skin. Seborrheic dermatitis in infants usually gets better by 6 to 12 months of age [34]. Dandruff usually persists into adulthood. In most cases, babies are not bothered by the symptoms of seborrheic dermatitis. Seborrheic dermatitis can be diagnosed by clinical findings without special diagnostic tests [36]. If seborrheic dermatitis is mild, topical antifungal creams or medicated shampoo containing ketoconazole, selenium sulfide, coal tar or zinc pyrithione, may be sufficient to control symptoms. If the child is not uncomfortable, parents are advised to leave mild seborrheic dermatitis untreated [34]. In more severe cases, topical steroids or topical calcineurin inhibitors can be prescribed to calm the inflammation. Oral antifungal medications may also be used.

### 2.6. Psoriasis

Psoriasis is now accepted as more than just a chronic inflammatory skin disease; rather, it is a chronic systemic inflammatory condition that primarily affects the skin but is related to various serious comorbidities that shouldn’t be overlooked. Therefore, a thorough understanding of all aspects of the disease in this age range is of utmost importance [35]. Psoriasis is relatively common, affecting between 1 and 3% of the population worldwide. One-third of psoriasis cases occur in childhood. Early recognition of the disease and taking appropriate approaches can help delay or even prevent significant impacts on a child’s quality of life or some comorbidities such as psoriatic arthritis (PsA) [35]. However, prevalence and clinical manifestations may differ from the those of the same type in adults. Among the variants of psoriasis, napkin psoriasis is particularly common in children under 2 years of age and is characterized by bright red to dull red erythematous plaques on the diaper area, and may be accompanied by more typical psoriasis in other locations. In a French epidemiologic study, napkin psoriasis was the most common (37.0%) form of psoriasis in infants [36]. Psoriasis in children can be categorized into several types. The categorization is mainly based on the shape and aspect of the lesions and sites of involvement. Most of the approved treatments for psoriasis in adults are the same as those used for children. However, most of them are not yet approved and require off-label prescription [35]. Studies regarding their safety and efficacy, particularly on long-term follow-up and outcomes, have not yet been conducted enough in this population. First-line topical therapies for all types of psoriasis include corticosteroids, vitamin D3 analogs, and calcineurin inhibitors, with keratolytic agents as adjuvants [37]. Corticosteroids are the most frequently used to reduce erythema, scaling, and pruritus acting with their anti-inflammatory, antipruritic, and antiproliferative properties. Calcipotriol and calcitriol are two vitamin D3 analogs that inhibit keratinocyte proliferation and induce differentiation [38]. They can be used as a monotherapy or in combination with a corticosteroid if it appears to be synergistic, as mentioned previously. Apart from safety and efficacy documented in the pediatric population, these agents also have a relatively low risk of adverse effects. Currently, tazarotene, anthralin, and coal tar are used as second-line topical therapies. Tazarotene, a topical retinoid, reduces keratinocyte proliferation and promotes differentiation, and is commonly used in combination with topical corticosteroids [37]. Although its safety and efficacy are not documented in children, it is approved for the treatment of psoriasis in adults. Anthralin has anti-inflammatory and antiproliferative proprieties and is effective and safe for use in children. It should be prescribed as a short-contact therapy to avoid skin irritation and pigmentation. As an effective and safe treatment for children, phototherapy, is primarily used for plaque or guttate psoriasis (that does not respond to topical therapy) with widespread involvement of the body, for debilitating palmo-plantar disease, and for patients who cannot receive systemic treatment [39]. There are no guidelines for systemic therapy for pediatric psoriasis, and data on the safety and efficacy of these drugs are limited. The most commonly used are acitretin, methotrexate, and cyclosporine, respectively, based on collected knowledge of their benefits and risks in children with other conditions, such as ichthyosis, juvenile rheumatoid arthritis, and organ transplantation [40]. It is also possible to combine these agents with topical drugs or phototherapy, or use in sequential or rotational strategy to maximize their benefits and reduce side effects. Biologics constitute attractive therapies for psoriasis. Most of them have also been recently approved for treatment in children. Biologics are very convenient when compared to other systemic therapies because of their better dosage regimens and fewer requirements for laboratory monitoring. As they are targeted therapies, they are much less likely to be toxic. However, serious complications such as opportunistic infection, malignancies, autoimmune diseases and tuberculosis reactivation have been reported in pediatric patients with juvenile inflammatory arthritis treated with these agents [36]. Biologic agents are considered second- or third-line agents and are restricted to severe and/or refractory cases of plaque, pustular, and erythrodermic psoriasis, or those with concomitant PsA. All patients should be screened for tuberculosis and laboratory studies before initiating treatment [41,42]. Although much is known already about pediatric psoriasis, standardized guidelines for the management and treatment of psoriasis in this age group are lacking and not yet approved [35].

### 2.7. Infectious Diseases

#### 2.7.1. Varicella (Chickenpox)

Varicella is the first manifestation of the varicella zoster virus (VZV) and often occurs in childhood. In one study, age-specific incidence rates were significantly different in a comparison of children and adults [43]. Among children 0 to 14 years of age, the incidence rates per 100,000 person-years in each age group were 11,000 for ages 0 to 14 years, and 175 for ages >20 years, despite a course of vaccination [43]. The pruritic polymorphous exanthema erupted and formed new effloresce ranging from maculae to papules, papulovesicles, vesicles, pustules, and crusts, which spread from the hairline to the caudal and lower extremities and typically involved the scalp and oral mucosa, which is a characteristic finding. The disease is accompanied by an increase in temperature and mild fatigue. Therapeutically, it is very important to prevent the secondary infections. This means symptomatic relief of itching by using a drying agent such as a tanning agent or lotio alba/calamine lotion with antihistamines. In severe cases, especially in immunocompromised children, antiviral drugs such as acyclovir, alternative famciclovir, and valacyclovir may be used [44].

#### 2.7.2. Pityriasis Rosea (Paraviral Exanthema)

Pityriasis rosea (PR) is a common exanthema and affects mainly adolescents. PR is asymptomatic, or can be mildly pruritic, but in approximately 25% of patients with PR, pruritus can be severe [45]. When PR is irritated by sweat, or tight clothes, the itching may be more aggravated. PR is a quite common condition in children, with an estimated annual incidence of 0.13%. Seasonal variation in the incidence of PR has been noted, which agrees with the possibility of a viral etiology of the condition. The condition is more common in the spring and winter seasons. Another point in favor of an infectious etiology for PR is the observation that a higher incidence (2%) has been documented in the developing world. The most common presenting age of PR is in children aged between 10 and 14 years, and young adults. The condition is rare in infants but has been described in very young infants aged three months old. Recent studies have pointed towards a possible slight female predominance with an estimated female-to-male ratio of 2:1. The disease is mainly associated with human herpes viruses 6 and 7 and shows a typical pattern: a primary medallion (Herald patch), followed by an exanthema along the skin creases (Langer lines) [46]. Primary and secondary lesions appear as ovular red-brownish plaques with typical collarette scaling. The exanthema is located mainly in the trunk and the proximal extremities. Patients show extremely sensitive skin, especially apparent after bathing, which can lead to eczema and severe itching [47]. Therefore, skin irritation such as vigorous washing and using too much soap should be avoided. The diagnosis is usually made clinically, but can be supported by the discovery of subacute dermatitis in histopathology of a skin biopsies. Blood tests for HHV6 (IgG or PCR) are not indicated because nearly 100% of individuals have been infected with the virus in childhood and conventional commercial tests do not measure HHV6 activity. Most patients will not require specific therapy as long as they follow the recommendations. In severe cases, various treatment options are available: H1-antihistamine for severe itching, a combination of mild topical glucocorticoids with low-dose UVB radiation, erythromycin, and acyclovir [48,49].

#### 2.7.3. Fungal Infections

Tinea is a fungal infection caused by dermatophytic fungi that invade the stratum corneum, hair shaft, and nail beds. The pruritus is an important and constant element of clinical manifestation of superficial dermatophytoses. However, studies that speculate on the intensity and prevalence of pruritus in pediatric patients with this disease are scarce. There is one cross-sectional study that has evaluated the prevalence and intensity of itch and superficial dermatophytosis in the Indian general population (16–74 years) [50]. In this study, the majority of patients had reported itch in the last three days (99%). According to a numeral rating scale (NRS), the mean intensity of the worst itch in the last three days was 6.8 ± 1.8 points. There is one study that investigated the pruritus of pityriasis versicolor; however, this is not a superficial dermatophytic disease. In this study, only 22% of patients reported itching sensation among 200 patients (15–60 years) with pityriasis versicolor [51]. In the two of the most common fungal organisms that cause tinea capitis are *Trichophyton tonsurans,* accountable for 95% of tinea capitis, and *Microsporum canis.* In the face, tinea corporis is caused by various fungal organisms, and tinea faciei is most often caused by *M. canis.* Lesions often appear as single or multiple rounded, scaly macules on the face. They often have raised border with typical erythema, and central pallor (or diminished pigmentation). Some children develop a butterfly-shaped patterns around the nasal areas, reminiscent of lupus. Lesions are variously itchy and may be hypopigmented or hyperpigmented. Diagnosis is made with a positive Wood lamp fluorescence of infected hair shaft, microscopic identification of hyphae and spores in the hair, or a scale sample taken from the edge of the lesion prepared with potassium hydroxide by microscopy. Identification of fungal organisms is made by culturing, which can take several weeks. Topical 2% miconazole, 1% clotrimazole, imidazole, cyclopyrroloxamine, or benzylamine is the first-line treatment for tinea. Treatment can take up to 4 weeks, and in some cases can take months to eradicate. Oral therapy with antihistamines and ketoconazole, itraconazole, terbinafine, or griseofulvin for pruritus is indicated for widespread infection or tinea capitis or cases resistant to topical treatment. Extensive disease or persistence despite oral therapy should lead clinicians to consider immunosuppression [52,53].

#### 2.7.4. Scabies

Scabies is very common in every age group. Scabies is endemic in many resource-poor tropical settings, with an estimated average prevalence of 5–13% in children [54]. After 2–3 weeks of a sensitization phase, in the primary infection, skin lesions appear on the predilected sites, which are the flexural surface of the wrist, axillary folds, and interdigital webs of the hands [2]. Typical lesions consist of papules and nodules and are related to a cellular immune response to *Sarcoptes scabiei* mites, eggs, and feces. The immune response is also what causes itching that is most severe at night [2,55]. Burrows in the skin, dug by female mites in the stratum corneum are pathognomonic. Patients in early infancy show facial and head involvement compared to older children and adults. Moreover, infants are more prone to developing polymorphic eczematous lesions with vesicles, papules, pustules, and scaling. In addition, young infants typically develop pustules in the plantar area. The standard therapy is 5% permethrin [55] and is recommended as a first-line therapy for patients older than two months of age. After successful treatment, some patients develop very itchy, reddish-brown papules/nodules, without the scabies mites. These are post-scabious granulomas, and respond to mild topical corticosteroid. In some cases, usage of emollients is sufficient [56].

#### 2.7.5. Pediculosis Capitis

Pediculosis capitis, caused by infection with *Pediculus humanus capitis*, is the most common form of lice and mainly affects school children [2]. Transmission occurs from head-to-head; transmission from other subjects via combs, brushes, or towels is rare. Pruritus is the most common symptom, which is due to a cellular immune response to salivary proteins of the louse. As the patients respond with scratching, the risk of bacterial infection with *Staphylococcus aureus* or *Streptococcus pyogenes* increases [2]. This can lead to impetiginization and cervical and suboccipital lymphadenopathy. A careful examination of the hair is necessary for an accurate and timely diagnosis. The therapy of choice involves topical pyrethroids such as permethrin or silicone oil. Recent European studies showed resistance of head lice to pyrethroids. However, molecular resistance (mutation of gene in the α-subunit of the voltage-gated sodium channel, the so-called knockdown resistance (kdr)-like gene) did not seem to correlate with failure using permethrin or pyrethrin in the treatment of pediculosis capitis in children. However, possible resistance should still be kept in mind [31,57].

### 2.8. Hereditary Disorders

Among the hereditary skin diseases such as ichthyosis, Darier disease, pityriasis rubra pilaris (juvenile familial type), hereditary angioedema, hyper IgE syndrome, Netherton syndrome (SPINK5 mutation), Olmsted syndrome (TRPV3 mutation), Alagille syndrome (NOTCH2 mutation) that occur in children, there are a variety of diseases that cause itching, and since they occur relatively rarely, little is known about them.

### 2.9. Dermatologic Disorders Due to Systemic Diseases or Drugs

Pruritus related to systemic diseases or events of adverse reaction to drugs are uncommon in childhood compared with adults. However, they do occur and involve the same organ systems as in adults (Table 2) [58]. The difference is that these diseases are typical childhood disorders that mainly include hereditary dermatoses. Table 2 provides an overview of the systemic diseases in childhood associated with pruritus. There are risk factors of drug eruptions and drug-induced pruritus, such as the female sex, immunosuppression, and certain genetic factors. Children with parents who have a true drug allergy are at a 15-fold relative risk for allergy reactions to the same drugs. In one 10-year observational study of children, cutaneous adverse reaction by drugs constituted about 20% of the adverse events, and most often (38.7%) from antimicrobial agents [59]. Differences in physiology and drugs that are frequently used in each group (children and adults) may also play a role in drug metabolism variances [60]. In children, there are drugs such as phenytoin, phenobarbital, carbamazepine, lamotrigine, allopurinol, sulfonamides, dapsone, minocycline, aspirin, vancomycin, azithromycin, abacavir, nevirapine, and Chinese medicines that could induce cutaneous adverse effects.

### 2.10. Neuropathic Pruritus and Postburn Pruritus

**Figure 3:**
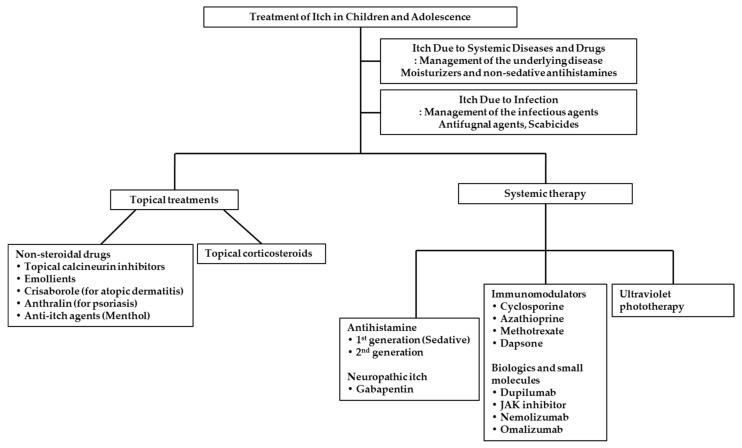
Therapeutic options for itching in children and adolescents.

Children recovering from a major burn injury often have a long-term complaint of itching, particularly during the wound healing process [61]. During this healing process, the pruritus can be prolonged for up to two years in some cases [62]. In the literature, the reported incidence of postburn pruritus ranges from 80% to 100% [63]. As noted, the Itch Assessment Scale Shriners, called the ‘Itch Man Scale’, has been validated for children and adolescents with a major burn injury [6]. The pathophysiology of pruritus is not completely understood to date; however, the pruritus and neuropathic pain share common pathways related to the afferent C-fibers to the spinothalamic tract [64]. At this point, the medications for neuropathic pain have been speculated upon in some of the literature [65]. The efficacy and safety of gabapentin and pregabalin in child burn survivors (≤5 years, 6–12 years, and ≥12 years) has been researched in a retrospective review of medical charts [66]. In this study, 91.4% of 112 children with postburn pruritus had a sufficient response. Additionally, 100% of 24 children treated with both pregabalin and gabapentin had an adequate response for pruritus. However, two children treated with gabapentin had the adverse effects of hyperactivity and sedation. One individual, with both medications, reported nausea, vomiting, and headaches, but this all resolved after the discontinuation of gabapentin. Thus, this study showed that gabapentin and pregabalin are effective and relatively safe in reducing postburn pruritus (Figure 3).

## 3. Treatments of Itch in Children and Adolescence 

### 3.1. Topical Treatments

#### 3.1.1. Emollient

Emollients should be suggested as the primary management strategy for localized pruritus, patients with eczema, such as atopic dermatitis, and xerosis in childhood [26]. Dry skin is caused by changes in epidermal lipid composition and increased transepidermal water loss. When the skin barrier function is compromised, hyperkeratosis, erythema, and itching occur. Moisturizers of mixed physiological skin lipids, similar to physiological skin lipids, are used to hydrate the stratum corneum, restore the barrier function, and relieve itching. Emollients may contain supplementary ingredients such as urea, polidocanol, menthol, or palmitoylethanolamide with anti-itch properties to target multiple components of the itch pathway [67]. However, in addition to the properties of the skin barrier, the transdermal absorption of drugs and topical formulations of emollients is related to the physical and chemical properties of the drug. Infant emollients should be free of fragrances, dyes, and preservatives, which are known to pose irritation and allergy risks. Emollients are generally non-antipruritic, but they lubricate and moisturize the skin, protect the integrity of the stratum corneum and skin barrier, and treat dry skin [68]. Emollients may be used as monotherapy for eczema in children or in combination with topical corticosteroids or calcineurin inhibitors. In clinical trials, regular application of emollient moisturizes and hydrates the skin, and helps the skin maintain its protective barrier effect as well as reducing the amount of topical corticosteroid needed for atopic eczema in all age groups. Through trials and long clinical experience, it has been proved that emollients are effective and safe for patients with eczema such as atopic dermatitis.

#### 3.1.2. Topical Corticosteroids

Topical corticosteroids are effective in treating a variety of skin inflammatory diseases, and reducing inflammation improves the associated itchiness. Especially in children, special care should be taken to balance the use of the lowest potency topical corticosteroids needed to achieve control so as to minimize potential side effects without undertreating inflammation [21]. In the case of using mid-potency to high-potency corticosteroids for flare-ups, a gradual titer reduction in potency is required to prevent rebound exacerbations [21]. Educating patients and families about the relative strengths, potential systemic and local side effects, and strategies for dose adjustment is critical to treatment success. Concerns of “steroid phobia” that can limit compliance should be openly discussed. As mentioned previously, aggressive skin hydration and the use of emollients are steroid-sparing and should be highlighted in discussions with the families. Choosing topical corticosteroid should be based on the severity of disease, its distribution, and the age of the patient. A discussion of the details of the application of topical corticosteroids and emollients is key to successful outcomes [21,69]. Topical corticosteroid must first be applied as a thin layer to areas of flare-ups. Topical emollient should be applied second in a thick layer on all unaffected areas of skin and should be avoided on areas already treated with topical steroids [69]. Providing sufficient amounts of topical corticosteroids is also important, paying attention to the severity of the disease.

#### 3.1.3. Topical Calcineurin Inhibitors

Topical calcineurin inhibitors (TCIs) are primarily used for inflammatory skin disorders such as atopic dermatitis and seborrheic dermatitis. The anti-inflammatory effects of topical calcineurin inhibitors result from selective blocking of cytokine transcription in activated T cells. In addition to their anti-inflammatory effects, they are thought to be effective in activating transient receptor potential vanilloid 1 (TRPV1) in peripheral C nerve fibers and reducing itching through subsequent desensitization [70]. The itching improves within 48 h after the first application and the itching continues to decrease with continued application. Initial stinging due to activation of TRPV1 is a common side effect, but stinging symptoms usually improve after repeated application over several days [70]. TCIs are preferred over steroids for long-term use because there is no side effect of skin atrophy even after long-term use in children [21]. Use of topical calcineurin inhibitors does not cause skin atrophy, making this class of drugs particularly effective in eyelid and facial dermatitis [21]. Calcineurin inhibitors may also be useful as a steroid-sparing agent for patients requiring long-term, anti-inflammatory treatment. Safety concerns arise from a small number of reported malignancies, animal toxicology studies, and the potential adverse effects (including immunosuppression and risk of lymphoma) observed in patients who received systemically administered calcineurin inhibitors to suppress solid organ transplant rejection [70]. Overall, TCIs have an established safety and efficacy profile as a long-term maintenance therapy in children with inflammatory skin disease [71].

#### 3.1.4. Small Molecules Drug

Phosphodiesterase-4 (PDE-4) is an intracellular enzyme and overactive in inflammatory cells of patients with AD. It has been targeted as a therapeutic plan to control the inflammation induced by AD [72]. Crisaborole 2% ointment inhibits PDE-4 dependent cyclic adenosine monophosphate degradation, resulting in the effectiveness in patients with mild-to-moderate AD [73]. The safety and efficacy of Crisaborole were evaluated by placebo-controlled trials. Crisaborole ointment was approved for use in children 2 years of age and older at first and extended the lower age limit from 24 months down to 3 months recently [73,74]. The approval for the expanded indication was supported by data from a Phase 4, open-label, clinical study showing that Crisaborole ointment was well-tolerated and demonstrated effectiveness in patients with mild-to-moderate AD [75].

### 3.2. Systemic Treatments

#### 3.2.1. H1-Antihistamines

H1-antihistamines are among the most commonly prescribed medicines in children. Indications include acute allergic reactions in pruritus, eczema, allergic skin disorders, and urticarial dermatitis [76]. H1 antihistamines are systemic drugs used as the first-line treatment for pruritus because of their relative safety, wide availability, and economics. However, data on the effectiveness of systemic antihistamines on pruritus are limited in the pediatric population. So far, data from randomized clinical studies have not demonstrated the effectiveness of antihistamines for diseases, other than urticarial [77]. Antihistamines include classic first-generation antihistamines and new second-generation antihistamines. Despite the longevity of their use, little is known about the pharmacokinetics and pharmacodynamics of first-generation antihistamines in infants and young children [78]. Among the first-generation antihistamines, hydroxyzine and diphenhydramine are approved for use in infants and children. Second-generation antihistamines have been studied more extensively in older children and adults, and for cetirizine, several studies have been conducted in younger children and infants down to 6 months of age. In addition, desloratadine, ebastine, and loratadine are approved for use in children aged 2 years and older, and azelastine, fexofenadine, and levocetirizine are available for use in children aged 6 years and older [76]. First-generation antihistamines easily cross the blood–brain barrier, causing sedation and anticholinergic side effects, which can cause severe discomfort [76]. Anticholinergic side effects include dry mouth, double vision, visual field disorders, and urinary discomfort. In addition, hydroxyzine is particularly lipophilic and may have a long half-life. Newer second-generation antihistamines are recommended as first-line therapy for most dermatologic diseases. These drugs are less sedative, have little anticholinergic activity, have fewer drug interactions, and require lower doses compared with first-generation drugs [76,77,78].

#### 3.2.2. Immunomodulators

Cyclosporine and azathioprine are effective for pediatric patients with inflammatory skin diseases such as atopic dermatitis, neurodermatitis, chronic urticaria, and autoimmune diseases that are hardly managed by antihistamines [79]. Cyclosporine is efficacious and acts rapidly in the majority of children with severe AD. Treatment with cyclosporine can provide sustained remission and seems to be well-tolerated in children; however, strict monitoring should be provided [80]. Side effects of cyclosporine are hypertension, infection, increased BUN/creatinine, or nephrotoxicity. Azathioprine can cause nausea, vomiting, anemia, and hypersensitivity: reactions include dizziness, diarrhea, fatigue, and skin rashes [68]. Mycophenolate mofetil has an immunosuppressive effect by specifically blocking lymphocyte proliferation and antibody production. In terms of safety, the incidence of toxicity is known to be lower than that of cyclosporine; however, the lack of controlled trials in children is a problem addressed by a change in the law on drug licensing a few years ago [71]. Clinical trials in children are essential to develop the best treatment strategies in a safe environment. Methotrexate has an anti-inflammatory effect on lymphocytes and neutrophils and is thought to be effective in treating itching. It is considered relatively safe in children compared to other immunomodulators [71]. It was shown to be effective in treating eczema and chronic urticarial; however, methotrexate has side effects such as nausea, vomiting, a sore mouth, skin rash, and thinning of the hair. Dapsone was reported to be effective in several types of chronic urticaria and angioedema. In using dapsone to treat a skin disorder, a physician might start on a low dose of dapsone and gradually adjust the treatment dose to control the skin disease [81]. Dosage is based on the patient’s medical condition and response to treatment. In children, the dosage is also based on age and weight. There are side effects such as dose-related anemia, peripheral neuropathy, photosensitivity, and methemoglobinemia. These are rare but serious side effects, requiring close monitoring. Since the standardized age of use of these immunomodulators has not been well established, additional studies for proving long-term safety would be helpful [71,81].

#### 3.2.3. Biologics and Small Molecules

Dupilumab is a fully human monoclonal antibody that blocks IL-4 and IL-13 in patients with AD. It has been shown to be effective in patients with severe atopic dermatitis and itching [82,83]. To analyze off-label use of dupilumab in children with AD, a multicenter retrospective review evaluated children prescribed dupilumab for moderate-to-severe AD [84]. About 90% of patients gained access to dupilumab after a mean of 9 weeks. This review supports dupilumab response and tolerability in most children and the safety is proven for use in children above 6 years [85]. Optimal dosing for patients under age 12 has not been defined, and availability in two different drug concentrations is still an important safety issue. Further studies on the schedules and dose adjustments for other pediatric skin diseases are in need [85].

Novel topical therapies such as phosphodiesterase-4 (PDE-4) inhibitors and Janus kinase (JAK) inhibitors are in clinical trials evaluating their efficacy and safety for the treatment of itchy skin diseases [73,74,75]. JAK signaling is involved in the signaling of AD related cytokines, such as IL-4, IL-13, IL-31, and IL-17 [86]. Several Janus kinase inhibitors are currently undergoing evaluation for their efficacy and safety in the treatment of AD. Recently, a 3-phase pivotal study of upadacitinib (JAK1 inhibitor) was conducted in pediatric AD patients over 12 years of age [87,88]. In the head-to-head results of upadacitinib (30 mg) and dupilumab, a more pronounced difference in response rate of upadacitinib was confirmed as EASI 90/100, as well as EASI 75 at week 16, the primary endpoint. In 2015, one small study by Levy et al. of tofacitinib (JAK1/JAK3 inhibitor) 5 mg once or twice daily as an add-on therapy to topical treatment in six patients with moderate-to-severe AD who had failed standard therapy showed promising results, with the average SCORAD index decreased by 54.8% at 14 weeks of treatment with tofacitinib. There were no serious adverse events [89]. In addition, tofacitinib, already approved for psoriatic arthritis by the U.S. Food and Drug Administration (FDA) since 2017, is the best studied JAK inhibitor in psoriasis [90]. The clinical trials for proving the safety and efficacy of upadacitinib and abrocitinib were conducted in patients over 12 years of age but have not yet been approved [91]. In contrast, baricitinib (JAK1/JAK2 inhibitor) was approved for treating atopic dermatitis in patients over 18 years of age [91,92,93]. A randomized, double blind, placebo-controlled study of 124 adults with moderate-to-severe AD treated with oral baricitinib included a 4 weeks TCS (triamcinolone 0.1%) run-in followed by 16 weeks of baricitinib, 2 or 4 mg, or placebo with continued use of TCS [93]. Baricitinib showed encouraging results in the only phase 2 study of the drug in moderate-to-severe psoriasis and 271 patients were randomized in five groups (baricitinib 2, 4, 8, 10 mg, and placebo). In this study, 43% of the patients in the 8 mg group and 54% in the 10 mg group achieved PASI 75 compared to 17% in the placebo group [90].

In addition, atopic dermatitis is characterized by a TH2-mediated immune response. Activated TH2 cells in patients have higher IL-31 levels and higher levels of IL-31 in skin. Nemolizumab, which blocks IL-31, markedly diminished pruritus within the first two weeks in patients with atopic dermatitis [94]. In this 16-week trial, the use of subcutaneous nemolizumab for patients with atopic dermatitis, including adolescents (above 12 years old), resulted in a greater reduction in pruritus than placebo plus topical agents. Longer and larger trials are necessary to determine whether nemolizumab has a durable effect and is safe for atopic dermatitis [94].

The recombinant human monoclonal IgG antibody omalizumab binds to free IgE and reduces the function of mast cells. One randomized clinical trial found that omalizumab significantly reduced atopic dermatitis severity and improved quality of life in a pediatric population with atopy and severe eczema despite highly elevated total IgE levels at baseline [95]. The result was associated with a potent topical corticosteroid sparing effect and may suggest that omalizumab is a treatment option for difficult-to-manage severe eczema in children with atopy. Safety and efficacy in patients with chronic urticaria have been proven in patients above 12 years of age. Apremilast (PDE4 inhibitor) regulates psoriatic pruritus by directing the production of inflammatory/non-inflammatory cytokines [96].

#### 3.2.4. Ultraviolet Phototherapy

NB-UVB is clinically effective and improves quality of life in children with moderate-to-severe eczema [97]. The effect is maintained for six months after treatment. In addition, it can be safely used by children with dermatitis and underlying illnesses and avoids drug interaction or compliance problems. In these cases, systemic drugs are difficult to use, whereas UV has few side effects other than a temporary sunburn-like reaction [98]. UV treatment is often the treatment of choice for pruritus if it is an indication.

### 3.3. Treatment for Itch Due to Systemic Diseases and Drugs

If a child’s itching is suspected to be due to a systemic disease, physicians should ask the child and caregiver to provide the patient’s detailed, systemic medical history and medications. Laboratory tests, such as kidney and liver function tests, should be performed and the patient should be checked for the presence of a causative disease. If present, the causative disease should be treated appropriately.

The most important thing in pruritus caused by systemic disease is the management of the underlying disease. For example, iron drugs are prescribed for iron deficiency anemia, and thyroid hormones for hypothyroidism. In the case of cholestatic pruritus, the biliary tract disease is treated first, and the itching can be controlled with cholestyramine. Moisturizers and non-sedative antihistamines may be prescribed as first-line treatments for all pruritus.

## 4. Conclusions

Itching in children and adolescents is mainly caused by skin diseases, and there are differences in the clinical characteristics of children and adults. Skin diseases in children may be misdiagnosed by other diseases, and if the diagnosis is delayed, there may be difficulties in the treatments. Physicians must take into account the special characteristics in childhood with respect to physiology, and pathophysiology, and also pharmacokinetics and pharmacodynamics that differ from those of adults. Although new treatments for some skin diseases and pruritus in children are being studied, treatments such as drugs used in adults are still limited in children, and further studies on there tolerability and safety are needed. Thus, further research is needed on the pathophysiology and treatment of itching in children.

## Figures and Tables

**Table 1:** Classification of skin diseases with itching in children and adolescents.

Age	Diagnosis	Frequency
Infancy (0–2)	Atopic dermatitis	25–50%
	Diaper dermatitis	12.2%
	Impetigo	6.6–6.9%
	Seborrheic dermatitis	3.6%
	Insect bites	2.4%
	Psoriasis	2.4%
	Papular urticaria	2.1%
	Viral exanthem	2.1%
Preschool (3–5)	Atopic dermatitis	24–35%
	Impetigo	2.3–7.1%
	Contact dermatitis	4.5%
	Insect bites	4%
	Urticaria	3.4%
	Seborrheic dermatitis	2.8%
	Papular urticaria	2.2%
	Psoriasis	2.2%
School (6–11)	Contact dermatitis	15.1%
	Atopic dermatitis	10–23%
	Acne vulgaris	13.0%
	Urticaria/Angioedema	2.9–4.9%
	Impetigo	4.7%
	Seborrheic dermatitis	4.1%
	Psoriasis	2.6–3.0%
	Xerosis	3.4%
Adolescence (12–16)	Acne vulgaris	8.9–25.2%
	Atopic dermatitis	4.4–27.4%
	Contact dermatitis	11.7%
	Seborrhic dermatitis	4.9%
	Pruritus	3.5%
	Psoriasis	3.0–3.1%
	Urticaria/Angioedema	3.0–5.4%
	Impetigo	2.6%

**Table 2:** Causes and characteristics of pruritus in various childhood diseases.

Classifications	Diagnosis	Clinical Manifestation of Pruritus
Metabolic disorders	Chronic renal failure (dialysis)	Accompanied by xerosis or prurigo Generalized or localized
	Liver diseases with or without cholestasis	Can be mechanically induced Not diminished by scratching Usually generalized pruritus
Endocrinal disorders	Thyroid diseases	Hyperthyroidism/hypothyroidism Associated with urticaria
	Diabetes mellitus	Secondary to diabetic neuropathy, Metabolic derangements associated with renal failure and xerosis
Infectious diseases	HIV	Itchy, red or purple, or painful
	Hepatitis C virus	Widespread, itchy, red rash
Hematologic disease	Iron deficiency	Generalized pruritus Skin lesion or irritation provokes scratching
	Polycythemia vera	After contact with water Accompanied by stinging sensation and prurigo Generalized pruritus
Paraneoplastic Diseases	Lymphomas and solid organ tumors	Premonitory onset Area of affected lymph nodes such as mediastinal sites
Hereditary dermotoses	Ichthyosis, Darier disease, pityriasis rubra pilaris (juvenile familial type), hereditary angioedema	-
Neuropathic pruritus	Postburn pruritus	
Drug-induced pruritus	Drug-induced pruritus Drug eruption	With or without skin rash Can occur after several months (lichenoid type)

## Data Availability

Not applicable.

## Abbreviations

AD	Atopic dermatitis
EASI	Eczema Area and Severity Index
IGA	Investigator global assessment
IL	interleukin
NB-UVB	Narrowband ultraviolet B
PsA	psoriatic arthritis
PR	Pityriasis rosea
TCIs	Topical calcineurin inhibitors
TRPV1	Transient receptor potential vanilloid 1
PDE-4	Phosphodiesterase-4
